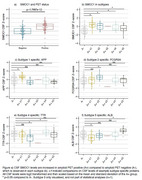# Alzheimer’s disease molecular subtypes in a clinical trial cohort

**DOI:** 10.1002/alz70859_105257

**Published:** 2025-12-25

**Authors:** Pieter Jelle Visser, Pallavi Sachdev, Satya Saxena, Charlotte E. Teunissen, Olav Mjaavatten, Frode Steingrimsen Berven, Betty M. Tijms

**Affiliations:** ^1^ Alzheimer Center Amsterdam, Neurology, Vrije Universiteit Amsterdam, Amsterdam UMC location VUmc, Amsterdam Netherlands; ^2^ Eisai Inc., Nutley, NJ USA; ^3^ University of Bergen, Bergen, Bergen Norway; ^4^ University of Bergen, Bergen Norway; ^5^ Alzheimer Center Amsterdam, Neurology, Vrije Universiteit Amsterdam, Amsterdam UMC location VUmc, Amsterdam, Noord‐Holland Netherlands

## Abstract

**Background:**

Alzheimer’s disease (AD) exhibits significant molecular heterogeneity, which poses challenges for treatment development. Understanding this heterogeneity is essential for creating more effective therapies and improving patient outcomes. Our recent research identified five distinct AD subtypes based on protein level alterations: hyperplasticity (s1), innate immune activation (s2), RNA dysregulation (s3), choroid plexus dysfunction (s4), and blood‐brain barrier dysfunction (s5). This study aims to determine if these subtypes can be identified in an independent cohort of early AD patients from a clinical trial.

**Method:**

Baseline cerebrospinal fluid (CSF) samples from 101 amyloid‐positive and 101 amyloid‐negative individuals (based on PET visual read) were collected from a Phase 3 program for elenbecestat in subjects with mild cognitive impairment (MCI) or mild dementia due to AD (NCT02956486). These samples were analyzed using 16‐plex tandem mass tag (TMT) mass spectrometry. Each individual was classified into an AD molecular subtype using a random forest classifier trained on our previous discovery cohort, utilizing 1249 overlapping proteins between the studies. We then compared CSF protein levels across the new sample for each subtype and against the amyloid‐negative group to assess amyloid specificity using linear regression models.

**Result:**

Amyloid‐positive individuals exhibited SMOC1 levels one standard deviation higher than amyloid PET‐negative individuals, confirming previous associations of SMOC1 with amyloid status (p < 0.01x10^‐10) (see figures a‐b). All five AD subtypes were identified in the amyloid‐positive group: 45 (44%) were classified as hyperplasticity s1; 20 (20%) as innate immune activation s2; 1 (1%) as RNA dysregulation s3; 18 (18%) as choroid plexus s4; and 17 (17%) as blood‐brain barrier dysfunction s5. Protein level comparisons across subtypes largely replicated our previous findings (see figures c‐f for example subtype‐specific proteins).

**Conclusion:**

The replication of the five AD molecular subtypes in an independent clinical trial cohort further supports the robustness of these subtypes and paves the way for personalized medicine and novel combination therapy approaches in AD.

**Reference**

1. Tijms, B. M. *et al*. Cerebrospinal fluid proteomics in patients with Alzheimer’s disease reveals five molecular subtypes with distinct genetic risk profiles. *Nat. Aging* 33–47 (2024) doi:10.1038/s43587‐023‐00550‐7.